# Impact of TiO_2_ Nanotubes’ Morphology on the Photocatalytic Degradation of Simazine Pollutant

**DOI:** 10.3390/ma11112066

**Published:** 2018-10-23

**Authors:** Syazwan Hanani Meriam Suhaimy, Chin Wei Lai, Hairul Anuar Tajuddin, Emy Marlina Samsudin, Mohd Rafie Johan

**Affiliations:** 1 Nanotechnology and Catalysis Research Centre (NANOCAT), Institute of Graduate Studies, University of Malaya, Kuala Lumpur 50603, Malaysia; hanani_ms@yahoo.com.my (S.H.M.S.); cwlai@um.edu.my (C.W.L.); emymarlinasamsudin@gmail.com (E.M.S.); 2 Department of Physics and Chemistry, Faculty of Applied Sciences and Technology, Pagoh Higher Education Hub, KM1 Jalan Panchor, Johor Bahru 86400, Malaysia; 3 Department of Chemistry, Faculty of Science, University of Malaya, Kuala Lumpur 50603, Malaysia; hairul@um.edu.my

**Keywords:** TiO_2_ nanotubes, photocatalyst, anodization, Simazine, photocatalytic, photodegradation mechanism

## Abstract

There are various approaches to enhancing the catalytic properties of TiO_2_, including modifying its morphology by altering the surface reactivity and surface area of the catalyst. In this study, the primary aim is to enhance the photocatalytic activity by changing the TiO_2_ nanotubes’ architecture. The highly ordered infrastructure is favorable for a better charge carrier transfer. It is well known that anodization affects TiO_2_ nanotubes’ structure by increasing the anodization duration which in turn influence the photocatalytic activity. The characterizations were conducted by FE-SEM (fiend emission scanning electron microscopy), XRD (X-ray diffraction), RAMAN (Raman spectroscopy), EDX (Energy dispersive X-ray spectroscopy), UV-Vis (Ultraviolet visible spectroscopy) and LCMS/MS/MS (liquid chromatography mass spectroscopy). We found that the morphological structure is affected by the anodization duration according to FE-SEM. The photocatalytic degradation shows a photodegradation rate of k = 0.0104 min^−1^. It is also found that a mineralization of Simazine by our prepared TiO_2_ nanotubes leads to the formation of cyanuric acid. We propose three Simazine photodegradation pathways with several intermediates identified.

## 1. Introduction

Pesticides, which can be further categorized into herbicides, insecticides and fungicides, are widely used in the agricultural industry to optimize the growth and production of crops. Simazine, in particular, is an effective herbicide applied for broad leaf and weed control but has resulted in serious public health issues in recent years [1]. The presence of toxic Simazine has been detected in several groundwater systems and thus compromised the quality of the drinking water system [2]. As the pollution of drinking water has brought serious negative impacts to human beings and living environments, photocatalysis has attracted much attention as a more feasible alternative to wastewater treatment techniques. Photocatalysis is based on semiconductor photochemistry, whereby most organic pollutants are decomposed into harmless substance using photons (light). Among the available nanostructured semiconductors, candidates such as TiO_2_, ZnO, WO_3_ [3], Bi_2_O_3_ [4], and SnO_2_ [5] have contributed to immense progress in various applications of TiO_2_ owing to the unique physical or chemical properties and high catalytic activity. Furthermore, TiO_2_ has versatile applications in the field of water splitting [6], dye-sensitized solar cells [7], gas sensors [8], photocatalytic [9] and photoelectric devices [10]. However, the effectiveness of its potential applications is restricted due to the fact that anatase TiO_2_ only absorbs UV (ultra violet) light corresponding to its band gap value of 3.2 eV. This resulted in low catalytic efficiency. There are various approaches to enhancing the catalytic properties of TiO_2_, including modifying its morphology by altering the surface reactivity and surface area of the catalyst. Different morphology alters the planes of TiO_2_ and makes it rich in active O 2p species as well. This morphology approach can be done via controlling the anodization duration during the fabrication of TiO_2_ nanotubes. Although a great deal of research has been devoted on optimizing anodization duration, it can be seen that it is developed for different applications. In recent years, several studies reported on the effect of anodization duration with respect to the photocatalytic degradation of methylene blue under UV (ultra violet) light irradiation [11], resistance towards corrosion [12], water splitting [13], etc. Just to cite one recent study, Regonini et al. [14] reported that well-defined nanotubes are grown at 10–20 min anodizing time and exhibit a maximum photoresponse value of 460 mA/cm^2^. According to Balakrishnan et al. [15], titanium anodized for 4.5 h gives the best formation to be applied in the human body with superior corrosion properties. The current research progress on the optimization of TiO_2_ nanotubes by anodization duration is summarized in Table 1. It was also observed that the optimum anodization duration depends on the experimental conditions and particular applications. In this study, the primary aim is to enhance photocatalytic activity by changing the TiO_2_ nanotubes’ architecture and orientation. A highly ordered infrastructure is favorable for better charge carrier transfer. It is well known that anodization affects TiO_2_ nanotubes’ surface properties by changing the nanotubes’ structure with the increasing anodization duration, which in turn influences the photocatalytic activity. Generally, the synthesis of TiO_2_ nanotubes on Ti foil is assisted by three main processes: (1) field-enhanced oxidation, (2) field-assisted dissolution, and (3) chemical dissolution. However, the disadvantages of this process are: it provides no limitations to nanotubes’ pore widening, and extensive dissolution happens over a long time, resulting in a loss of the nanotube structure [16]. In other words, prolonging the duration could damage the nanotubes’ structure. So, finding the optimum anodization duration is important due to the fact that porous nanotubes’ structures provide a number of active sites and further increase the ability of the photocatalyst to degrade pollutants. Most photocatalytic degradation activity also focused less on Simazine organic pollutant removal application. Therefore, it would be interesting to focus on optimizing the anodization duration to enhance the photocatalytic degradation of Simazine. Although the optimization of TiO_2_ nanotubes by anodization duration has been studied intensively, to our knowledge, there is limited information available on Simazine removal via photocatalysis. Thus, the specific objective is to study the effect of TiO_2_ anodization duration and corresponding nanotube morphology on its efficiency in the removal of Simazine via photocatalysis. The morphologies of TiO_2_ nanotubes are also further optimized to enhance the photocatalytic degradation.

## 2. Methodology

### 2.1. Materials and Catalyst Preparation Method

Ti foils with a 0.127 mm thickness, 99.7% metal basis purity (1 mm × 5 mm) from Sigma-Aldrich (St. Louis, MO, USA) were used as photoelectrodes. Prior to anodization, Ti foils were washed with distilled water and acetone for 30 min and dried in air. The electrochemical anodization treatment was carried out using direct current power supply with the distance between cathodic (Ti foil) and anodic (Pt rod) of about 2 cm for all samples. A mixture of 0.5 wt% ammonium fluoride (NH_4_F), 100 mL ethylene glycol (EG) and 1.6 wt% potassium hydroxide (KOH) was used as electrolytes. Anodization was performed at different durations of 30 s, 10 min, 20 min, and 60 min. After the anodization process was complete, the prepared samples were rinsed in distilled water, followed by acetone, and subsequently dried in air. Annealing was then performed at 450 °C for 2 h in air with the purpose of improving the oxide crystallinity and anatase transformation [11]. For comparison with photocatalytic activity, a TiO_2_ nanotube sample was prepared without a heat treatment process.

### 2.2. Catalyst Characterizations

The morphological changes of the formed nanotubes were examined using scanning electron microscopy (SEM, FEI Quanta 200, Hillsboro, OR, USA). The elemental compositions were measured using energy dispersive X-ray spectroscopy (INCA Software v. 4.15). X-ray diffraction (XRD, Bruker D8 Advance, Karlsruhe, Germany) with Cu Kα radiation was used to study the crystallinity phase of the catalysts. The photoluminescence (PL) and Raman spectra were recorded using InVia Raman Microscope (Renishaw, Gloucester, UK) at a laser wavelength of 514 nm and laser power of 50% to observe its charge carrier behavior and chemical structure.

### 2.3. Photocatalytic Activity Evaluation

**Figure 1:**
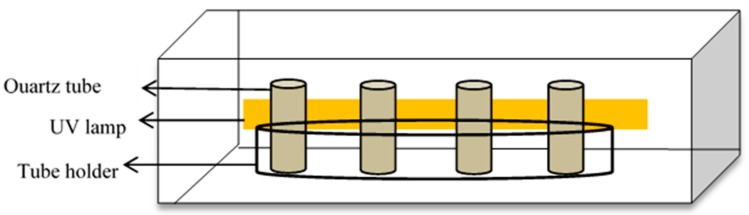
The photocatalytic reactor used in this study.

The photocatalytic degradation of Simazine was evaluated using the prepared TiO_2_ nanotubes under UV light irradiation. Simazine was purchased from Fluka (Buchs, Switzerland) and used without further purification. 50 mL of Simazine (concentration 1.0 ppm) solutions were placed in a reactor across from a UV irradiation source (Figure 1). The source of light was a 95 W UV lamp. Each TiO_2_ nanotubes of 1 mm × 5 mm dimension was immersed in Simazine solution and put in the dark to ensure the adsorption-desorption of the Simazine on the TiO_2_ nanotube surface. During UV irradiation, 3 mL Simazine solution was taken at 60-min time intervals. After irradiation was complete, the percentage of Simazine degradation was measured by UV-Vis spectroscopy (Varian Cary 50 Series, Agilent Technologies, Santa Clara, CA, USA). LCMS/MS Q-TOF (Agilent 6550 iFUNNEL, Agilent Technologies, Santa Clara, CA, USA) was used to identify the intermediate products produced during photocatalytic degradation. The mobile phase used was 50%:50% *v*/*v* H_2_O (deionized water): acetonitrile. The analysis was conducted at a flow rate of 1.0 μL/min and the wavelength was set to 222 nm. A 20 μL sample was injected into C-18 reversed-phase column (4.6 mm ID (inner diameter) × 250 mm, 5 μm) for liquid chromatography separation.

## 3. Results and Discussion

### 3.1. SEM Analysis

There are various approaches to enhancing the catalytic properties of TiO_2_ nanotubes, including reducing the band-edge positions, promoting the separation of e-h pairs, and modifying the morphology by altering the surface reactivity and surface area of the catalyst. Accordingly, the primary aim of this study is to enhance the photocatalytic activity of TiO_2_ nanotubes by altering their architecture; charge carrier transfer is more favorable in highly-ordered TiO_2_ nanotube arrays. It is also worth mentioning that anodic oxidation duration could affect the TiO_2_ nanotubes’ surface properties, which in turn influences the photocatalytic activity of TiO_2_, by changing the structure of the TiO_2_ nanotubes. Taking these facts into account, we must optimize the anodic oxidation duration with the aim of increasing the number of active sites in TiO_2_ and enhancing their photocatalytic abilities in photodegrading the pollutants.

**Figure 2:**
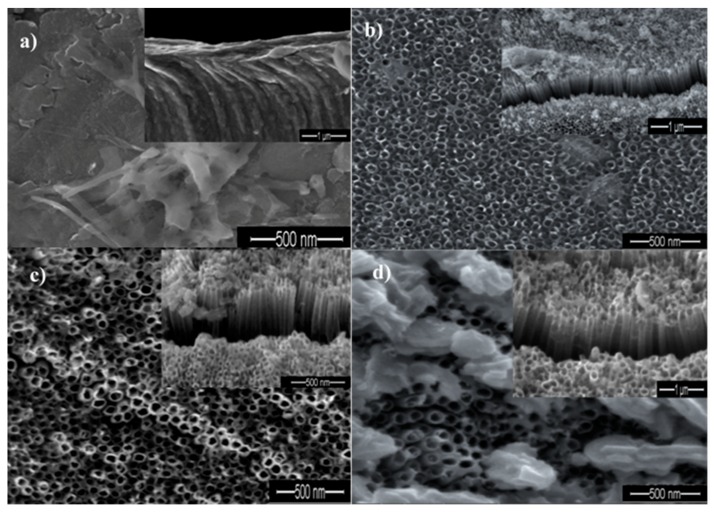
SEM images of TiO_2_ nanotubes fabricated by the anodization of Ti foil at 30 V for different times: (**a**) 30 s; (**b**) 10 min; (**c**) 20 min and (**d**) 60 min.

Figure 2a–d show the field emission scanning electron microscopy (FE-SEM) images of oxide layers on Ti sheets anodized at 30 s, 10 min, 20 min, and 60 min, respectively. It is clear that the structure and morphology of the oxide layers on Ti sheets change when changing the anodic oxidation durations. It is also observed that, in Figure 2a, TiO_2_ does not exist in a tube form if the exposure time is short (~30 s). In a cross- section, there is also no apparent barrier within the tubes. These observations give us a hint that a compact oxide layer with a porous structure does not form at this stage. According to Li et al. [19], no nanotubes could be formed within a relatively short period during the anodizing process since the Ti just starts to be oxidized to Ti^4+^ ions at the surface layer of the Ti foil. During this stage Ti^4+^ ions increased and the surface layer of Ti foil was removed. Based on their findings, TiO_2_ nanotubes can only be observed after 10 min of exposure time. The resulting TiO_2_ nanotubes were found to be debris-free due to the relatively high number of ejected Ti^4+^ ions during the chemical dissolution of the electrolyte over a relatively long anodizing time [19]. Figure 2b shows that a dense, aligned, compact and smooth wall of nanotubes could be obtained after 10 min anodic oxidation. It is interesting to note that nanotubes with an irregular porous layer could be obtained by further increasing the anodization time to 20 min. This is evident from the TiO_2_ nanotubes showing yellowish colors (Figure 2c). Accordingly, the light yellowish region indicates the presence of shorter tubes, while the dark yellowish region indicates the presence of longer tubes [20]. Based on these observations, TiO_2_ nanotubes with irregular structures could be formed after 20 min exposure time. Specifically, the anodic oxidation of Ti involves the chemical reaction of Ti^4+^ ions with oxygen in the electrolyte. Electric field dissolution is then induced under applied potential, forming pores and eventually producing nanotubes. It is noteworthy that field-assisted oxidation and dissolution involves the formation of an oxide layer and the dissolution of that oxide. According to the field-assisted oxidation and dissolution growth mechanism, the pores generated can continuously and consistently grow into tubes due to the continuation of chemical dissolution and electrochemical oxidation during this period of time. By prolonging the anodizing time to 60 min, a layer of flower-shaped structures forms and covers the top layer of the TiO_2_ nanotubes’ surface. This phenomenon was probably due to an over-dissolution reaction occurring between the oxide layer and Ti metal interface [16]. Yurddaskal et al. [17] also claimed that a non-homogeneous pore structure on TiO_2_ could be formed by increasing the anodic oxidation duration, considering the nanotubular structure of TiO_2_ has been destroyed [17]. Based on these findings, it could be concluded that anodic oxidation duration (time) plays a critical role in influencing the surface features and the morphology of TiO_2_ nanotubes.

### 3.2. Elemental Analysis

Table 2 tabulates the elemental composition of the TiO_2_ nanotubes prepared at varied anodizing durations. It is noteworthy that energy dispersive X-ray spectroscopy (EDX) is restricted to the analysis of elemental composition on selected region only, thus does not represent the total weight and atomic concentrations in TiO_2_ nanotubes. The weight percentage of the titanium (Ti) and oxygen in the optimized anodic oxidized specimen (10 min) were identified as 62.59% and 37.41%, respectively. It is clear that the atomic percent of Ti is inconsistent, but there is a significant decrease in Ti atomic percentage in the 20 min anodic oxidized sample due to the formation of an irregular TiO_2_ structure. Moreover, the percentage of oxygen elements also increased when increasing the anodization duration. These results imply that the increase in anodizing time will influence the morphology of the TiO_2_, causing a change in the total Ti and O composition. Based on the elemental composition results, it was also determined that a relatively large proportion of nanotubes consists of Ti and O elements. This gives a hint that the compound formed is TiO_2_. It is also noteworthy that long anodization duration will facilitate the formation of a thick oxide layer due to the extraction of hydroxyl ions or oxygen from the electrolyte (strong oxidation).

### 3.3. Physical Appearance

**Figure 3:**
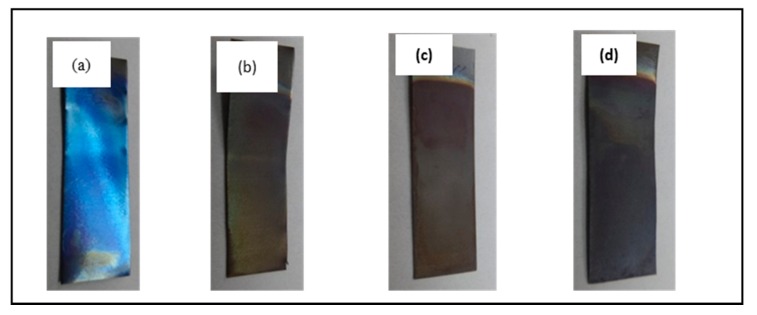
Digital images of physical appearance of Ti foil anodized at (**a**) 30 s; (**b**) 10 min; (**c**) 20 min; and (**d**) 60 min.

Figure 3 illustrates that the physical appearance of the anodized substrates (after 30 s anodic oxidation) is bluish, while the 10 min, 20 min, and 60 min anodized samples are yellowish. The bluish color of the 30 s anodized sample is attributed to the oxide layer formed on the surface of the substrate at the initial stage of anodic oxidation. Taking into account that the chemical-dissolution process is limited to 30 s of anodic oxidation, this restricted the formation of uniform nanotubes as confirmed by the field emission scanning electron microscopy (FE-SEM) image (Figure 2a). Interestingly, increasing the anodic oxidation duration changed it from bluish to yellowish. However, there was no significant difference in color among the 10, 20 and 60 min anodized samples. The appearance of a yellow spot on these 10, 20 and 60 min anodized samples indicated the formation of an oxide layer on the Ti substrate since a longer anodic oxidation duration allows for a sufficient chemical dissolution process to take place, triggering a greater change in the nanotubes’ structure.

### 3.4. XRD Analysis

**Figure 4:**
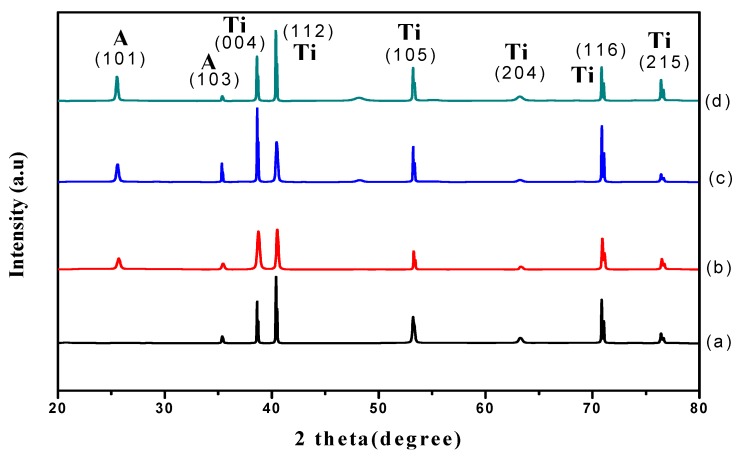
XRD patterns of TiO_2_ grown at 30 V and annealed at 450 °C for 2 h with anodizing time of (**a**) 30 s; (**b**) 10 min; (**c**) 20 min and (**d**) 60 min.

Figure 4 shows the XRD patterns for samples anodized for varied durations of time. Accordingly, only those samples anodized for 30 s showed the titanium metal phase (Figure 4a) and the peak for the 30 s sample is ascribed to the Ti substrate. Interestingly, the diffraction peaks for samples anodized at 10 min, 20 min, and 60min showed a predominant polycrystalline anatase phase at 25.7°, 37.5°, 38.4°, 39.2°, 54.8°, 63.7°, 70.0° and 76.4° corresponding to (101), (103), (004), (112), (105), (204), (116), and (215) plane orientations, (ICSD-01-075-1537). These results are in good agreement with the results of a previous study [21]. All samples were found to have a tetragonal crystal system associated with the distortion of TiO_2_ lattice during phase transformation [22]. Additionally, it was found that the (101) anatase peak intensity increased when increasing the anodic oxidation duration. The increase in crystallinity is probably associated with the formation of a bigger anatase crystallite size at a longer anodic oxidation duration. Debye-Scherer’s equation was then used in the present study to estimate the average crystallite size based on the anatase (101) diffraction peaks. It is clear that a shorter anodic oxidation process (30 s) induced the nucleation of anatase TiO_2_ with a crystallite size of 8.5 nm. The relatively small crystallite size obtained at 30 s indicated that a short anodic oxidation duration retards the crystallite growth. The crystallite size increased to 25.9, 34.5 and 41.6 nm after being subjected to 10 min, 20 min and 60 min anodic oxidation, respectively. These findings were confirmed by the presence of sharp and intense peaks at a relatively long anodic oxidation duration. It is noteworthy that the growth of TiO_2_ nanotubes when increasing the anodizing time is in good agreement with Li et al. [19]. A possible explanation for the growth of TiO_2_ nanotubes with the increase in anodizing time is that the anodization duration played a role in influencing the phase transformation of the crystal growth.

### 3.5. Raman Analysis

**Figure 5:**
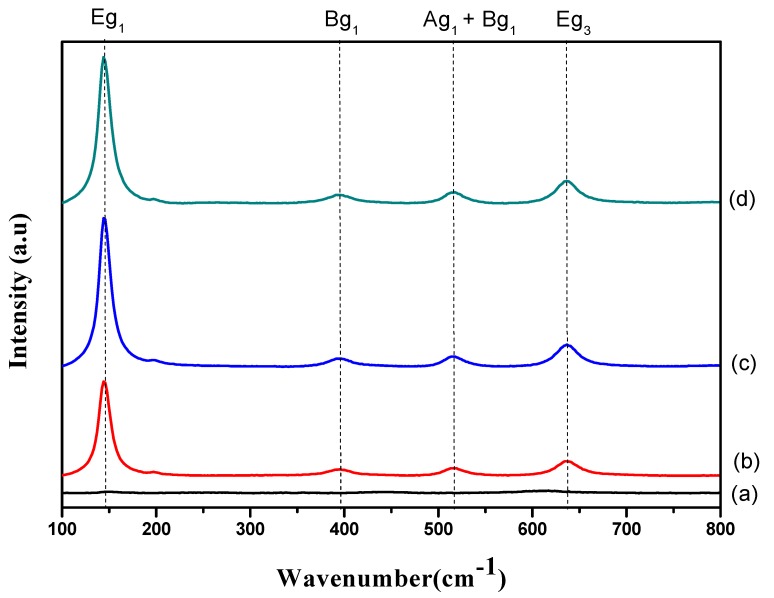
Raman spectra of (**a**) 30 s, (**b**) 10 min, (**c**) 20 min and (**d**) 60 min samples.

The influence of varied exposure times on the formation of TiO_2_ nanotubes was further examined using Raman spectroscopy (Figure 5). It is clear that all samples exhibited a similar pattern, except those with an exposure time of 30 s. Specifically, five Raman peaks at frequencies of 135, 196, 395, 516, and 637 cm^−1^ (corresponding to the anatase phase of TiO_2_) were detected [19,23,24]. However, there was a variation in the crystallinities of the nanotubes with the sample anodized at 30 s, as shown in Figure 5a. This is due to the fact that only an oxide layer is formed during this stage. The absence of these five anatase phonon modes for the sample anodized at 30 s manifests the presence of the amorphous phase. Additionally, these crystallites’ evolution implies that the anodic oxidation duration has a significant effect on the crystalline structure of nanotubes. Apart from that, the peak intensities (Figure 5b–d) increased when increasing the anodic oxidation duration. However, band broadening was observed with a longer exposure time. The Eg mode redshift confirmed the increase in crystallite size, in line with the XRD results.

### 3.6. Photoluminescence (PL) Analysis

**Figure 6:**
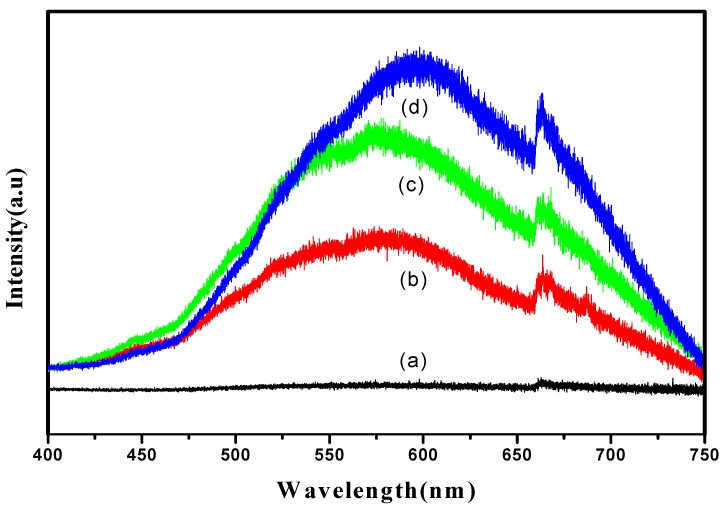
PL spectra of (**a**) 30 s; (**b**) 10 min; (**c**) 20 min and (**d**) 60 min samples.

Photoluminescence spectra (PL) analysis was conducted in order to investigate the separation efficiency of the charge carrier for all samples (Figure 6). It can be clearly seen that the emission spectra of all samples show a similar broad characteristic luminescence peak with dominant emission in the range of 550 nm to 700 nm. However, no typical peak emission was detected for the sample produced at 30 s, as shown in Figure 6a. PL emission mainly results from the recombination of photogenerated charge carriers. No peak emission occurring at 30 s might be due to less oxide formation, thus an inability to facilitate the transfer of the charge carrier. In addition, it can be clearly seen that the PL emission intensities increased as the anodization duration increased. The sample produced at 10 min showed the lowest PL intensity compared to the 20 and 60 min samples. A reduction in emission intensity is attributed to a highly ordered TiO_2_ layer formed at 10 min, demonstrating an effective electron and holes separation. This is in good agreement with the FE-SEM images in Figure 2. The higher intensity of the peak corresponds to a higher recombination rate. TiO_2_ nanotubes produced at 60 min showed the highest PL emission intensity because the destroyed structure of TiO_2_ will favor the recombination. The PL results suggested that the charge carrier mobility is affected differently by the TiO_2_ nanotubes’ structures. In other words, a good morphological structure of nanotubes would efficiently transport the charge carrier, lowering electron and hole recombination.

### 3.7. Photocatalytic Degradation Analysis

**Figure 7:**
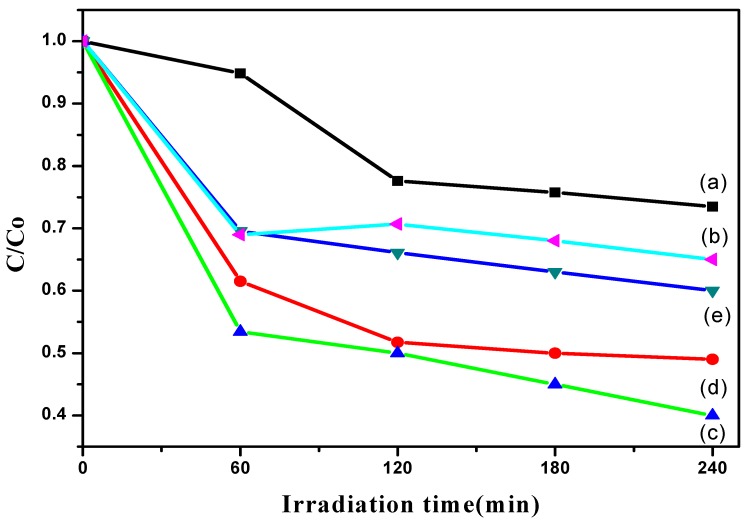
Plot of the C/Co vs. irradiation time of (**a**) TiO_2_ without heat treatment and heat-treated TiO_2_ nanotubes anodized at an anodization time of (**b**) 30 s; (**c**) 10 min; (**d**) 20 min and (**e**) 60 min.

The photocatalytic activity of TiO_2_ nanotubes was examined by measuring the ability of TiO_2_ nanotubes to photodegrade the model organic pollutant Simazine. In the photocatalytic activity principle, initial photon absorption occurs when the energy is equal to or greater than the band gap of the semiconductor photocatalyst. As a consequence, electrons get excited from valence band (VB) to conduction band (CB), generating electron-hole pairs and forming holes at the VB. The resulting holes tend to oxidize organic molecules or combine with water to form hydroxyl radical (·OH), a super oxidant. Meanwhile, a reaction at CB is reduction of oxygen, O_2_ to superoxide radical anion (·O^2−^). The generated radicals have high potential to degrade Simazine in water sources. However, electron-hole pair recombination limits the efficiency of photodegradation. Based on the mechanism, the photodegradation efficiency is highly dependent on the photocatalyst. Figure 7 shows the photocatalytic activity of TiO_2_ without heat treatment and heat-treated TiO_2_ nanotubes anodized at different anodization times. It can be seen that the concentration of Simazine was different for all photocatalysts as the irradiation time increased under all synthesis conditions. TiO_2_ without heat treatment showed low photoactivity (Figure 7a). As reported in most studies, TiO_2_ is known to be in amorphous structure without undergoing a thermal annealing process [11,25]. In contrast, the crystallinity of the TiO_2_ nanotubes is enhanced, resulting in the improvement of photodegradation after a heat treatment process. Moreover, this result shows that the anodic oxidation duration has an effect on the photodegradation ability. The reaction rate was calculated based on Equation (1), where C_0_ is the initial concentration and C is the Simazine concentration (ppm or mg/L) at time t:

(1) $$\mathrm{kt}=\ln(\frac{C_{0}}{C}),$$

A 10 min anodizing time showed the best photodegradation rate, following a pseudo-first order (k = 0.0104 min^−1^) with 60% of Simazine degradation percentage, which may be attributed to the existence of dense, highly ordered nanotubes. The highly ordered alignments are proved by the FE-SEM results, in which TiO_2_ nanotubes are perpendicular to the titanium substrate. This provides a channel for efficient charge transfer and results in the increment of active sites of photocatalysis compared to a non-oriented structure. It is a key for efficient photodegradation. When the number of active sites for absorption increases, the photodegradation capability increases because there are many reactions taking place. This indicates that the photocatalytic activity is driven by the structural morphologies, particularly a well-aligned structure, which helps provide a direct electron transfer pathway. However, extending the anodizing time to 60 min caused a flower-shaped structure of TiO_2_ tubes, as shown in the FE-SEM results. This led to a decrease in the photodegradation rate (k = 0.0061 min^−1^). The flower-shaped structure decreased the photodegradation performance because fewer active sites were present on the photocatalyst’s surface. Therefore, the amount of hydroxyl radicals and superoxide anions on the TiO_2_ nanotubes’ surface was insufficient to degrade the Simazine.

**Figure 8:**
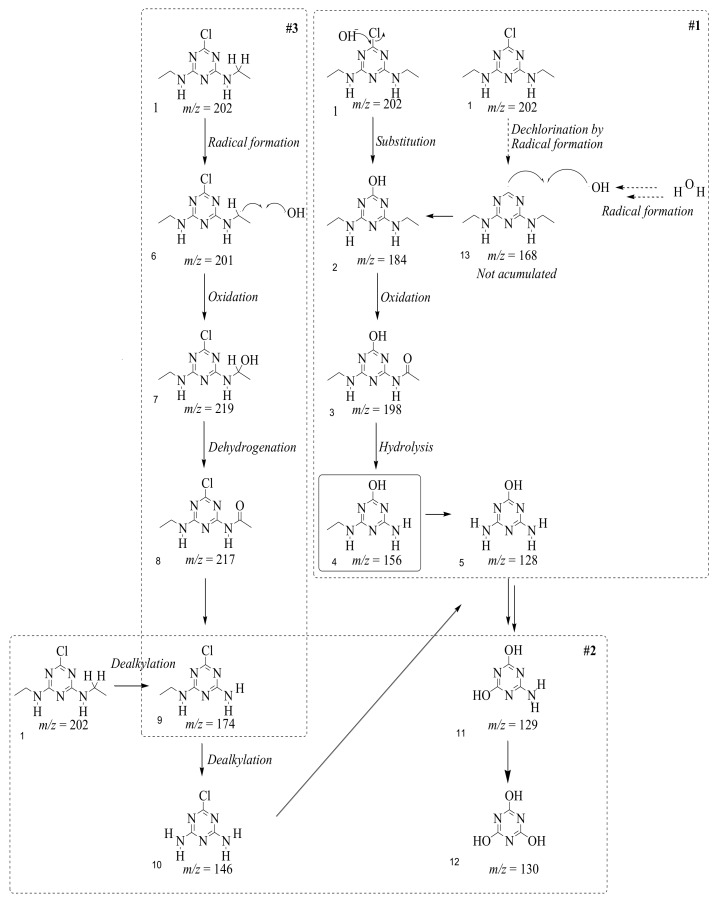
Proposed degradation pathways of Simazine.

In this study, TiO_2_ was prepared in a 10 min anodization time as a photocatalyst to degrade Simazine at a 1.0 ppm concentration. LCMS/MS/MS analysis was used to analyze the different intermediates produced during the photocatalytic degradation of Simazine within 240 min. Various intermediates are identified based on their mass measurements of ions (*m/z* value) in a positive mode. The *m/z* values of all compounds are listed in Table 3. Before degradation, the LCMS/MS/MS analysis clearly identified the presence of Simazine at *m/z* = 202. In general, there are three pathways proposed and the degradation scheme of Simazine is shown in Figure 8.

The first proposed degradation [26] that occurred in the first pathway begins with the substitution of a hydroxyl for a chloro group and dechlorination through radical formation. A molecular ion with *m/z* of 184 corresponding to C_7_H_12_N_5_OH was formed. Accordingly, oxidation took place at the ethyl group while the hydroxyl group was unaffected, giving rise to compound 3 with *m/z* of 198. The progress of further mineralization showed the presence of products 4, 5, 11 and 12.

In the second pathway [26], dealkylation of amines was taken into account due to the presence of compounds 9 and 10. This path continued by the ·OH radical attacked at –C–Cl by a substitution process, changing to an –OH group. The intermediate was identified as compound 5. Furthermore, this product experienced the loss of –NH_2_, and cleavage into a *m/z* = 129 intermediate. The resulting intermediate could be further attacked by the hydroxyl group to generate stable cyanuric acid (*m/z* = 130). Overall, it was found that the photodegradation process leads to the formation of cyanuric acid.

Pathway #1 and #2 have been proposed by Wei Chu in 2009 [26]. In addition to that, we also proposed a new pathway which labelled as #3 in Figure 8. In the third pathway, the major reaction of Simazine photodegradation is first initiated by the formation of a radical attacking ethyl group instead of hydroxyl. Mineralization by this route yields to the formation of seven different compounds. In the early stage of mineralization, intermediate number 6 with *m/z* of 201 was expected to form. However, this intermediate is not stable and can easily react with moisture. The generation of an intermediate with *m/z* of 219 occurred after ·OH radical attack on the C by one of the ethyl groups forming compound 7. Then, compound 8 with an amide moiety (*m/z* of 217) was produced through oxidation (or dehydrogenation), followed by a pathway similar to the first pathway until cyanuric acid (*m/z* = 130) is formed.

## 4. Conclusions

Our study reveals that:

(1) Prolonging the anodization time can engineer the structure or nanotubes, thereby significantly influencing their morphology and crystallinity. At a very short anodization time (30 s), no dense nanotubes were observed on the substrates. With elongated oxidation duration, a uniform oxide layer grew. However, too long an oxidation time resulted in damage because F^−^ ions existing in electrolyte can erode the nanotubes. The optimum anodization time was found to be 10 min in this study.
(2) The photodegradation of 1.0 ppm Simazine was successfully achieved by a TiO_2_ nanotubes photocatalyst. Interestingly, three photodegradation pathways are proposed in this study and non-toxic cyanuric acid is found to be the final product. LCMS/MS/MS analysis was used to identify the intermediates produced during the mineralization process. Identification of various intermediates will be beneficial for future advanced oxidation and water treatment processes.

## Figures and Tables

**Table 1:** Past research on anodization duration parameter.

Experimental Condition	Major Observation	References
Anodization duration: 5 min~2 h Electrolyte: 75 mL of EG + 3 mL of H_2_O + 0.3 g of NH_4_F Voltage: 30 V Annealing temp: 450 °C	Optimum anodization time: 20 min Extended anodizing time (1~2 h) causes some damage to the nanotubes Application: The incident photon to current efficiency (IPCE)	[14]
Anodization duration: 15 min, 30 min, 60 min, 120 min, 240 min. Electrolyte: Diff concentration of HF (0.25% HF, 0.5% HF, 1.0% HF, 2.0% HF) Voltage: 30 V Annealing temp: 500 °C	Optimum anodization time: 240 min The photocatalytic activity of the sample anodized for 240 min was the highest of all samples. Application: Degradation of methylene blue (MB) under UV light	[17]
Anodization duration: 0.5, 1, 2 and 4.5 h Electrolyte: 1 M Na_2_SO_4_ + 0.5 wt% NaF Voltage: 20 V Annealing temp: 600 °C	Optimum anodization time: 4.5 h Titanium anodized for 4.5 h is the best candidate for use in the human body due to its lower corrosion rate and absence of localized corrosion. Application: Corrosion resistance ability	[15]
Anodization duration: 1, 3 and 9 h Electrolyte: Set 1 (Glycerol 75%, H_2_O 25% and [F^−^] = 0.14 M) Set 2 (EG 98%, H_2_O 2%, [F^−^] = 0.14 M) Voltage: 20 V Annealing temp: 400 °C	Optimum anodization time: 9 h Anodization time appears much more effective at electrodes grown in ethylene glycol than at those grown in glycerol. Application: Water splitting	[18]

**Table 2:** Energy-dispersive X-ray spectroscopy (EDX) spectra of annealed TiO_2_ at 450 °C for 2 h.

Anodizing Duration	Element	Weight %	Atomic %
30 s	O	25.21	50.23
	Ti	74.79	49.77
10 min	O	37.41	64.16
	Ti	62.59	35.84
20 min	O	33.32	59.94
	Ti	66.68	40.06
60 min	O	38.36	65.07
	Ti	61.64	34.93

**Table 3:** Compounds detected during Simazine photocatalytic degradation.

No	Compound Name	Structural Formula	Molecular Formula	Molecular Weight (*m/z*)
1	6-chloro-*N*,*N*′-diethyl-1,3,5-triazine-2,4-diamine	C_7_H_12_ClN_5_	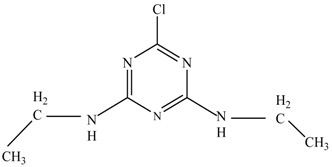	202.0871
2	6-hydroxy-N,N0-diethyl-1,3,5-triazine-2,4-diamine	C_7_H_12_N_5_OH	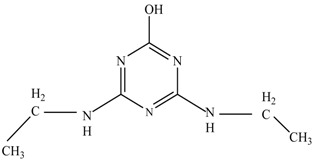	184.1194
3	6-hydroxy-2-acetamido-4-ethylamino-1,3,5-triazine	C_6_H_10_N_5_COOH	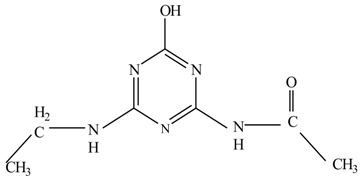	198.0987
4	6-hydroxy-2-amino-4-ethylamino-1,3,5-triazine	C_5_H_8_N_5_OH	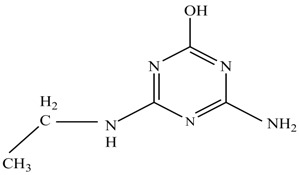	156.0884
5	6-hydroxy-2,4-diamino-1,3,5-triazine	C_3_H_4_N_5_OH	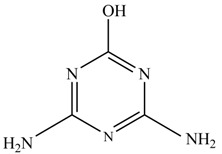	128.0569
6	6-chloro-N2,N4-diethyl-1,3,5-triazine-2,4-diamine radical	C_7_H_11_ClN_5_	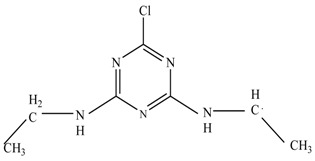	201.5509
7	1-((4-chloro-6-(ethylamino)-1,3,5-triazine-2-yl)amino)ethanol	C_7_H_11_ClN_5_OH	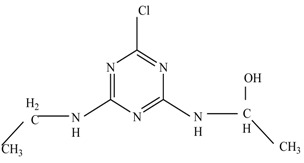	219.3340
8	*N*-(4-chloro-6-ethylamino-[1,3,5]triazine-2-yl)-acetamide	C_7_H_11_ClN_5_O	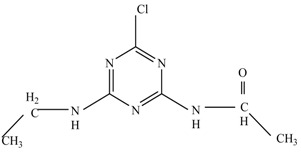	217.0124
9	6-chloro-*N*-ethyl-[1,3,5]triazine-2,4-diamine	C_5_H_8_N_5_Cl	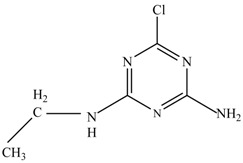	174.1572
10	6-chloro-[1,3,5]triazine-2,4-diamine	C_3_H_4_N_5_Cl	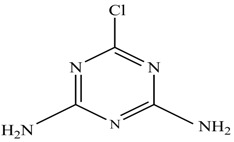	146.0225
11	2,6-dihydroxy-4-amino-1,3,5-triazine	C_3_H_2_N_4_(OH)_2_	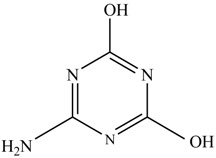	128.5669
12	2,4,6-trihydroxy-1,3,5-triazine	C_3_N_3_(OH)_3_	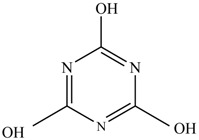	130.1596
13	*N*,*N*′-diethyl-1,3,5-triazine radical	C_7_H_12_ClN_5_	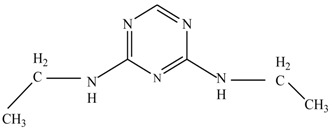	166.5097
